# Traditional and Complementary Medicine Use During Postpartum Period: A Cross-Sectional Analysis at a Rural, Public Maternal and Child Health Clinic in West Malaysia

**DOI:** 10.7759/cureus.15410

**Published:** 2021-06-03

**Authors:** Mohd Hafiz Ridzuan, Mohd Fairuz Ali, Chai-Eng Tan, Aznida Firzah Abdul Aziz

**Affiliations:** 1 Family Medicine, Bagan Serai Health Clinic, Bagan Serai, MYS; 2 Department of Family Medicine, Faculty of Medicine, Universiti Kebangsaan Malaysia Medical Centre, Kuala Lumpur, MYS

**Keywords:** traditional medicine, complementary medicine, post-partum

## Abstract

Background and objective

Despite the widespread use of traditional and complementary medicine (TCM) during pregnancy, very few studies have focused on the use of these practices during the postpartum period among women in Malaysia. This study aimed to evaluate users’ profiles and factors associated with the use of TCM during the postpartum period among women attending a community clinic in rural Malaysia.

Materials and methods

A cross-sectional study was conducted among 210 women at a maternal and child health clinic in Bagan Serai, Perak, Malaysia from January to April 2019. A self-administered questionnaire was used to determine the prevalence, the different types, and reasons for the use of TCM and perceptions toward TCM. Factors associated with TCM use during the postpartum period were derived from multiple logistic regression analyses.

Results

The prevalence of TCM use during the postpartum period was 66.2% among the subjects. The most common type of TCM used was massage (88.3%), and the most common reason reported was to improve general well-being (72.1%). “Family belief” had the highest mean for influence toward TCM use (mean: 3.63). Malays (adj. OR: 4.52, 95% CI: 1.93-10.59, p=0.001) and those having a low monthly household income (adj. OR: 3.68, 95% CI: 1.24-10.91, p=0.019) were the groups that were more likely to use TCM.

Conclusion

TCM use during the postpartum period is highly prevalent among Malay women with low household monthly incomes. Further studies should be conducted to address the benefits and risks of using TCM during the postpartum period.

## Introduction

The use of traditional and complementary medicine (TCM) is steadily gaining in popularity not only in Malaysia but also in other parts of the world [1]. In a nationwide study conducted among the general population in Malaysia, 69.4% were found to have used TCM at least once in their lifetime, and 55.6% reported using TCM in the past 12 months [2]. This indicates that TCM is a well-accepted practice among Malaysians.

The World Health Organization (WHO) has recognized TCM as an integration of two concepts: traditional medicine and alternative or complementary medicine. Traditional medicine refers to culture-based health practices, knowledge, and skills used for maintaining health, as well as the management of physical and mental illnesses. Complementary medicine, on the other hand, refers to healthcare practices that are not part of the mainstream healthcare system and are not indigenous to the culture of the population [3].

Recognizing the importance of TCM in our population, the Malaysian Ministry of Health has set up a Traditional and Complementary Medicine Division in 2004. The national policy on TCM is in alignment with the WHO TCM strategy [3]. The Malaysian Ministry of Health has defined TCM as a form of health-related practice designed to prevent, treat and/or manage illnesses, and/or preserve the mental and physical well-being of individuals [4]. Both definitions concur that TCM practices are not part of mainstream medicine but have a supplementary role [3].

There are many types of TCM and different studies have resorted to different methods of categorization. In Malaysia, TCM is categorized based on local ethnic practices. According to the TCM division of the Malaysian Ministry of Health, TCM is classified into the following six groups: (1) traditional Malay medicine, (2) traditional Chinese medicine, (3) traditional Indian medicine, (4) homeopathy, (5) complementary medicine, and (6) Islamic medical practices [4]. This classification may leave out some practices of TCM in East Malaysia where there are many more diverse elements of ethnic and cultural practices.

In studies from the United States (US) and many other foreign countries, TCM is classified into five categories, which are (1) mind-body practices (e.g., yoga, tai chi), (2) biologically-based (e.g., diet and dietary supplements), (3) manipulative/movement-based practices (e.g., chiropractic, massage), (4) whole medical system and traditional healers (e.g., homeopathy, naturopathy), and (5) energy medicine [5]. There is insufficient published research to determine the exact breakdown of the types of TCM as practiced by Malaysians. We have found a local study conducted in Eastern Malaysia that reported that Malay herbs were the most commonly used TCM medicine followed by traditional Chinese herbs, while reflexology was the most popular complementary medicine modality [6].

According to the published literature, factors that influence the use of TCM are mostly sociodemographic, such as gender, age, income levels, education [1,2]. Generally, older age groups are more aware and show a preference toward the use of TCM over conventional medicine due to its perceived efficacy and safety, i.e., absence of adverse effects [7]. However, studies that have focused on perceptions toward TCM use are scarce.

The postpartum stage refers to the period immediately following childbirth until six weeks post-delivery. During this period, women undergo various physiological changes before returning to their pre-pregnancy state, and they experience various kinds of difficulties such as physical, emotional, psychological, and social problems, which may encourage them to try TCM therapies [5]. Symptoms that have been identified to drive TCM usage include urinary incontinence, perineal pain, fatigue, depression, sexual problems, and bowel problems [8]. This trend has also been observed in the First World countries such as the US, where around one-third of postpartum women have reported using TCM during their postpartum period for various reasons such as low back pain, self-reported anxiety/depression, sleep problems, and also to support breastfeeding [5].

However, the use of TCM is not devoid of side effects. The use of herbs has been shown to cause allergic reactions, drug interactions, and an increase in the risk of bleeding [9]. Some herbal preparations are associated with potential carcinogenic properties or may cause organ toxicity resulting in hepatitis, nephropathy, and cardiomyopathy [10]. Adulterated traditional medications may contain steroids, which can be detrimental to health with prolonged use [11]. Acupuncture carries the risks of infections and mechanical injuries, leading to pneumothorax and spinal injury [10]. There have also been reports regarding adverse effects associated with massages, such as disc herniation, soft tissue trauma, and other injuries, which are most likely related to spinal manipulations [12]. In light of these factors, there are some legitimate concerns about certain TCM practices and medications, particularly those prescribed by unqualified or unregulated practitioners.

Some studies on the use of TCM among postpartum women have focused on the use of herbs [13-16]. Herbs are also reportedly used during labor to ease and shorten the process [13]. About 13.9% of women have reported consuming herbs during pregnancy, and 52.9% have ingested herbs during the postpartum period, despite a documented report of a higher incidence of neonatal jaundice in women taking herbs during pregnancy [14]. Previous studies have also found that factors that are most commonly associated with TCM use during the postpartum period are educational levels [15,16], age [17], primiparous status, normal spontaneous delivery, and breastfeeding [16].

Taking these factors into account, we believe more information is needed before some regulatory measures regarding the use of TCM can be implemented, especially with respect to women during their postpartum period. This study provides vital information on the different methods of TCM used among postpartum women and discusses the various factors associated with its use.

## Materials and methods

This cross-sectional study was conducted among postpartum women who attended maternal and child health services at the Bagan Serai Health Clinic, a rural public primary health clinic in Northern Malaysia, from January 2 to April 30, 2019. This primary health clinic provides healthcare services to the local population of 43,520 people, which mainly consists of Malays, followed by Chinese, Indian, and other minority ethnic groups.

The inclusion criteria for enrolment were as follows: postpartum mothers, who were in the period from delivery to six months post-delivery, Malaysians, and able to read and understand English or Bahasa Melayu. Those who did not give consent and did not fulfill the inclusion criteria were excluded from this study.

A convenience sampling method was used for data collection. The sample size was calculated using the formula for finite population by Krejcie & Morgan and based on the prevalence of TCM use among postpartum women (28%) from a study by Birdee et al. in 2014 [5]. A minimum sample of 209 participants was needed to reach a precision of 0.05 with a 95% confidence level, with an additional 10% factored in for non-complete responses.

Study instrument

A set of bilingual self-administered (in Bahasa Melayu and English) questionnaires were distributed to the participants during their postnatal visits at the clinic every Wednesday during the study period. The questionnaire consisted of 28 questions, which were divided into three sections. The first section collected information on sociodemographic data and characteristics, which consisted of age, ethnicity, educational level, marital status, employment status, estimated monthly household income, parity, breastfeeding status, and comorbidities. Only those who had used or were currently using TCM during the postpartum period were requested to answer section two, which comprised queries related to details on TCM usage, reasons for using TCM, and degree of influence to use TCM. Section 3 collected information about perceptions toward TCM.

The second section of the questionnaire (i.e., usage of TCM) was a descriptive survey form adapted from a study by Ali et al. [18]. This section gathered information regarding the types of TCM, as well as onset, duration, and frequency of using TCM. For the reasons of using TCM and degree of influence to use TCM, this part relied on the study by Tam et al. [7]. Responses regarding the degree of influence were to be recorded on a scale of 1-5 (where 1 stood for "not influential" and 5 meant "very influential").

The third section (perceptions toward TCM) was adapted from Tam et al. [7], and it was intended to measure participants’ level of agreement regarding the safety of TCM, the efficacy of TCM, level of trust toward TCM, health awareness, and their feelings after using TCM. Based on Tam et al. [7], "safety" was defined as the perception that TCM was unlikely to cause danger, while "efficacy" was defined as the ability of TCM to produce the desired effect. "Trust" was the belief in the reliability of TCM, whereas "health awareness" was defined as the perception toward health. Meanwhile, "feeling" was defined as perceived beneficial effects after using TCM.

The final questionnaire was subjected to face and content validation processes and was pretested on 20 women including colleagues, staff members, and patients at the clinic for consistency and understanding. We found that the internal consistency was acceptable with a Cronbach α range of 0.724-0.873. Responses were to be documented using a five-item Likert scale to indicate the level of agreement or disagreement with each statement (where 1 stood for "strongly disagree" and 5 meant "strongly agree"). The mean score reflected the users’ perception toward TCM, with a higher score indicating a stronger level of agreement. The mean score was calculated for each item. The questionnaire was given to each participant along with a cover letter and informed consent form during their visit to the maternal and child health clinic.

Data analysis 

All statistical analyses were performed using SPSS Statistics version 22 (IBM, Armonk, NY). The descriptive analysis summarized the patients’ sociodemographic and clinical profiles. Bivariate and multivariate logistic regression analyses were used to determine the association of sociodemographic and clinical profiles with TCM use. A p-value of <0.05 was considered statistically significant.

## Results

Sociodemographic characteristics of the subjects

A total of 210 women were invited to participate in this study. While four declined to participate, another 11 were excluded as they were non-Malaysian (n=6) and were unable to communicate in either Bahasa Melayu or English (n=5). The final analysis was conducted among 195 participants. The response rate for this study was 92.8%.

Table 1 summarizes the sociodemographic characteristics of the women who participated in the study. Malays constituted the ethnicity with the highest number of participants (84.6%), and the majority of them were in the 20-39 age group (96.4%). The mean age of the participants was 30.3 years (SD: 5.1). An assessment of comorbidities was performed, which showed that the majority of the women had no comorbidities during recruitment (91.8%). Of all women who participated in this study, 129 (66.2%) had used TCM during their postpartum period.

**Table 1:** Sociodemographic characteristics (n=195) SD: standard deviation; RM: Malaysian ringgit

Variables	Categories	N (%)	Mean (SD)
Ethnicity	Malay	165 (84.6)	
	Chinese	8 (4.1)	
	Indian	21 (10.8)	
	Others	1 (0.5)	
Age, years	20-29	92 (47.2)	30.28 (5.18)
	30-39	96 (49.2)	
	≥40	7 (3.6)	
Employment status	Employed	81 (41.5)	
	Unemployed	114 (58.5)	
Household income level	≤RM 3,000	159 (81.5)	
	≥RM 3,001	36 (18.5)	
Educational level	No formal education	1 (0.5)	
	Primary	7 (3.6)	
	Secondary	132 (67.7)	
	Tertiary	55 (28.2)	
Parity	Primiparous	79 (40.5)	
	Multiparous	116 (59.5)	
Breastfeeding status	Yes	131 (67.2)	
	No	64 (32.8)	
Comorbidity	Yes	16 (8.2)	
	No	179 (91.8)	
Use of traditional and complementary medicine	Yes	129 (66.2)	
	No	66 (33.8)	

TCM use among women during the postpartum period

Table 2 shows the characteristics of women who used TCM during their postpartum period. Our findings showed that Malays (91.4%) were the ethnic group with the highest number of TCM users during the postpartum period. The majority of these women were in the younger age group of 20-39 years (96.9%). Most women had at least a secondary education level (63.5%), were unemployed (55%), and had a household income of less than RM 3,000 (76%). More than half of the women were multiparous (58.1%) and breastfed their babies (67.4%).

**Table 2:** Traditional and complementary medicine use among women during the postpartum period (n=129) SD: standard deviation; RM: Malaysian ringgit

Variables	Categories	N (%)
Age, years; mean (SD): 30.22 (0.45)	20-29	62 (48.1)
	30-39	63 (48.8)
	≥40	4 (3.1)
Ethnicity	Malay	118 (91.4)
	Chinese	5 (3.9)
	Indian	6 (4.7)
	Others	0 (0)
Educational level	No formal education	1 (0.8)
	Primary	3 (2.3)
	Secondary	82 (63.5)
	Tertiary	43 (33.3)
Household income level	≤RM 3,000	98 (76.0)
	≥RM 3,001	31 (24.0)
Employment status	Employed	58 (45.0)
	Unemployed	71 (55.0)
Parity	Primiparous	54 (41.9)
	Multiparous	75 (58.1)
Breastfeeding	Yes	87 (67.4)
	No	42 (32.6)

Details of TCM use among women during the postpartum period

**Figure 1:**
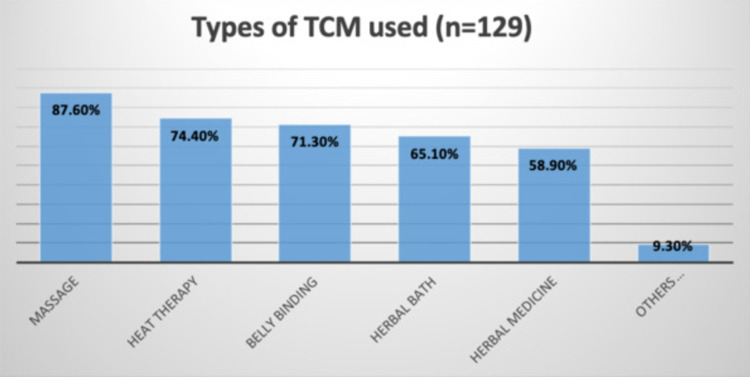
Types of TCM Used TCM: traditional and complementary medicine

**Figure 2:**
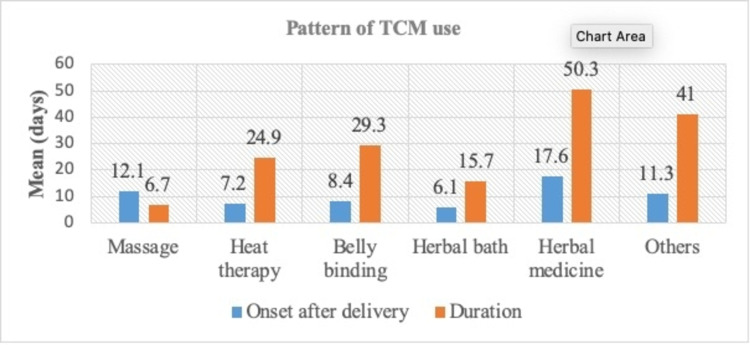
Pattern of TCM use among postpartum women TCM: traditional and complementary medicine

Our findings showed that the most common type of TCM used was massage (87.6%), followed by heat therapy (74.4%), belly binding (71.3%), herbal bath (65.1%), herbal medicine (58.9%), and others (9.3%), which included dieting programs, vitamins or supplements, and sauna bathing (Figure 1). Participants reported starting to take herbal baths six days after delivery on average, heat therapy by day seven, and belly binding by day eight. The TCM method with the shortest duration was massage, which was only practiced for six days on average, while the longest duration was associated with oral medicine consumption (mean: 50.3 days) (Figure 2). Other TCM (diet/vitamins/sauna) were reportedly used frequently: ~36 times per month (while massages were used only five times per month) (Table 3).

**Table 3:** Pattern of TCM use among postpartum women TCM: traditional and complementary medicine

Types of TCM	Onset after delivery (days, mean)	Duration (days, mean)	Frequency of use (times/month)
Massage	12.1	6.7	5.2
Heat therapy	7.2	24.9	27.9
Belly binding	8.4	29.3	30.8
Herbal bath	6.1	15.7	19.4
Herbal medicine	17.6	50.3	33.0
Others	11.3	41.0	36.3

The reasons for using TCM during the postpartum period were queried. Most of the women used TCM to improve their general well-being (72.1%). Half of the women said they took TCM to enhance breastfeeding (51.1%) and one-third used it for losing weight (34.9%); 27% used TCM due to cultural beliefs and 24% due to back pain. Some women took TCM for sleep problems (6.9%) and self-reported anxiety/depression (3.1%).

Regarding the degree of influence to use TCM, family beliefs exerted the highest level of influence among the women to use TCM (mean: 3.63, SD: 1.25). This was followed by personal choice (mean: 3.61, SD: 1.19). Other factors such as TCM providers (mean: 2.68, SD: 1.28), friends (mean: 2.66, SD: 1.21), and the media (mean: 2.59, SD: 1.21) had a moderate level of influence on TCM use among postpartum women.

As for the perceptions toward TCM among the users, respondents had a high level of awareness and significantly favorable attitude toward a healthy diet (mean: 3.96, SD: 0.84), followed by a positive feeling after using TCM (mean: 3.38, SD: 0.66). They also expressed the view that TCM was safe (mean: 3.16, SD: 0.73) and efficacious (mean: 3.07, SD: 0.64). However, their belief in the reliability of TCM (trust) was low (mean: 2.88, SD: 0.73).

Factors associated with TCM use among women during the postpartum period

In univariate analysis, ethnicity, education level, household income, and employment status were shown to be significantly associated with the use of TCM (p<0.25). All these four variables were further subjected to multivariate logistic regression analysis.

The findings showed that the Malay ethnicity (p=0.001) and having a low monthly household income of RM 3,000 and below (p=0.019) were independently associated with the use of TCM during the postpartum period. Malays were 4.5 times (adj. OR: 4.52, 95% CI: 1.93-10.59) and those with a low monthly household income were 3.6 times (adj. OR: 3.68, 95% CI: 1.24-10.91) more likely to use TCM during the postpartum period (Table 4).

**Table 4:** Factors associated with the use of TCM among women during the postpartum period (n=195) TCM: traditional and complementary medicine; OR: odds ratio; CI: confidence interval; RM: Malaysian ringgit

Variables		TCM		Simple logistic regression analysis		Multiple logistic regression analysis	
		Nonuser	User	P-value	Crude OR (95% CI)	P-value	Adjusted OR (95% CI)
Age group	20-29 years	30	62	0.582	1.55 (0.33-7.37)		
	30-39 years	33	63	0.651	1.43 (0.30-6.78)		
	≥40 years	3	4		1.00		
Ethnicity	Malay	47	118	<0.001	4.33 (1.91-9.81)	0.001	4.52 (1.93-10.59)
	Non-Malay	19	11		1.00		1.00
Educational level	Secondary	52	86	0.028	2.25 (1.09-4.64)	0.312	1.52 (0.67-3.45)
	Tertiary	12	43		1.00		1.00
Estimated monthly income	≤RM 3,000	61	98	0.008	3.86 (1.42-10.46)	0.019	3.68 (1.24-10.91)
	>RM 3,000	5	31		1.00		1.00
Occupational status	Employed	43	71	0.176	1.53 (0.83-2.82)	0.245	1.48 (0.76-2.88)
	Unemployed	23	58		1.00		1.00
Parity	Nulliparous	25	54	0.592	1.18 (0.64-2.17)		
	Multiparous	41	75		1.00		
Comorbidity	Yes	60	119	0.747	1.19 (0.41-3.43)		
	No	6	10		1.00		

## Discussion

This study confirms that TCM usage among women during the postpartum period in rural areas was high, with about two-thirds (66.2%) of women using either massages, heat therapy, belly binding, herbal bath, or herbal medicine. These findings echo a previous study in Kedah, which found that 54.7% of postpartum women used TCM practices [19]. However, some other studies have reported a lower prevalence of TCM use, and this could be attributed to variations in the definitions of TCM, the types of TCM modalities included in the surveys, location, and cultural differences [5,14,15].

Many cultures around the world observe specific rituals during the postpartum period. The rituals differ in different countries and among different ethnic groups [20]. In Malaysia, various ethnic groups have their own culturally specific postpartum practices, and the period is commonly referred to as "berpantang" or "confinement period". Malay women commonly practice "bengkung" (the traditional body wrap, belly binding, or girdle), postnatal massage, "jamu" (traditionally prepared supplements/herbs) hot compresses ("bertungku"), and herbal bath during their postpartum period [21,22]. Chinese women undergo a 30-day "Zuo yuezi" period (confinement period) after delivery when they practice various steps believed to restore bodily balance, including dietary modifications, consumption of herbs, herbal baths, and certain cultural taboos. During this period, mothers have the opportunity to recuperate, rest, and concentrate on the baby for breastfeeding [21-23]. Indian mothers usually practice herbal baths, postnatal massage, nourishing herbal-based diets, and belly binding [21,22]. The Indian postnatal care practice is mainly based on Ayurvedic methods [24]. Considering the wide variation in TCM practices among various ethnic groups, some cross-cultural assimilation of practices is likely to occur, and hence it is worthwhile to explore the use of TCM beyond the consumption of herbs among Malaysian women.

The TCM of choice among this population was overwhelmingly massage. This is not surprising because the majority of the participants were Malay, and traditional Malay massage is considered to be an important part of the care regimen during the confinement period [21]; it is similar to the practice of TCM in Singapore [25]. The view among Malaysian women is that massage can improve blood circulation and provide therapeutic heat to the whole body [21]. The use of certain TCM practices during the postpartum period has been shown to have some benefits. For example, acupressure massages can result in faster recovery to the original body composition after childbirth and help to reduce fatigue and stress [26]. Topical application of St. John’s wort on Cesarean scar facilitates wound healing and minimizes the chances of inflammation and scar formation [27]. A case report on the use of Malay massage in a stroke patient during the postpartum period has reported that it appeared to positively influence stroke recovery [28]. However, massage and other forms of manipulation and body-based therapies are not as popular among postpartum women in the US [5], where mind-body practices appear to be the most popular TCM modality.

In terms of age, the majority of women who used TCM during postpartum were below 40 years old. This could be due to the higher representation of this age group in our cohort due to their higher fertility rate. Regarding the onset and duration of TCM use, a majority of the women started using TCM during the first month after delivery and used them for a period of fewer than 30 days. This corresponds with the confinement period of Malay women who generally confine for 40-44 days, while Chinese and Indian women usually confine for 30 days [21]. Postpartum confinement involves a set of practices meant to assist the mothers in their recovery from the effects of pregnancy and childbirth. Hence, the practices that one adheres to during this stage may have the potential to promote recovery and prevent future ill-health [21].

This study showed that the most common reason for using TCM in this population was a desire to improve general well-being, followed by an impetus to support breastfeeding. This finding is similar to that of a local study that found that postpartum women took herbs for promoting general health and energy [14]. Herbs have been used for centuries as galactagogues to improve lactation and increase milk supply, but scarce data exist regarding its safety and effect on newborn health, which can be an indication that the incidence rate is low [29]. 

This study found that the selection of TCM modality was predominantly influenced by the respondents’ family beliefs, followed by personal choice. In Asian culture, families in general and elder family members in particular exert a strong influence on caring and management practices or rituals during the postpartum period. This finding aligns with that of Chang et al. (2015), but their study was conducted among the general population in Sabah [6]. Based on our findings, when a woman experienced distressing symptoms or emotional instability, she would tend to seek TCM for treatment and prevention. Participants in this study also agreed that they themselves had a high level of health awareness regarding dietary practices. A possible explanation for this is that users who have stronger health awareness would choose a TCM modality that allowed them to take control of their own health needs [7]. This result supports a previous study, which observed that health awareness among women reinforced the TCM practice [30].

We have observed that postpartum women of Malay ethnicity were more likely to use TCM during the postpartum period when compared to their non-Malay counterparts. This could be attributed to the large number of Malay respondents who reside in this rural area. The finding can also be explained by the fact that only a small number of non-Malays participated in this study. Chinese postpartum practices commonly include a special dietary practice to restore the balance between "hot and cold" [21]. Chinese women would consume "hot" foods such as ginger, wine, and rice while avoiding "cold" foods such as vegetables and fruits [23]. Dietary modification is a common practice among postpartum women of various different races, including Malay and Indian women [21]. However, they may or may not include herbs in their food. Hence, some participants may not have interpreted this as part of TCM. This could also explain the differences in related findings between Malay and non-Malay women.

Women from households with a low monthly income are more likely to use TCM during the postpartum period. This could be explained by looking at the most commonly used types of TCM, which are massage, heat therapy, and belly binding. In Malaysia, these forms of TCM are less expensive, more accessible, and very popular in rural areas, whereas in big cities, TCM services are highly commercialized and hence tend to cost more. This finding is similar to that of a previous study, which showed that the majority of TCM users had a household income of less than RM 3,000 per month [19]. It can also be postulated that mothers from lower-income households normally share accommodation with their parents or in-laws, which may lead to the exertion of stronger levels of influence and values on their part in mothers' decision to use TCM; however, we did not specifically look into this aspect in our study.

We believe this study adds to the existing body of knowledge about TCM use among postpartum women in rural Malaysia. However, there are some limitations to this study. The method used was convenience sampling, and therefore our findings and inferences may not be generalizable to the broader population. Also, this study was conducted at a single center in a rural area in the northern part of West Malaysia, and hence the findings should be interpreted cautiously as it may not be an accurate reflection of the views of the entire Malaysian population, especially since it did not take into account practices in East Malaysia. Potential recall bias among the participants is another major limitation. However, this was limited by restricting the inclusion criteria to women who had delivered in the preceding six months. The possibility of response bias should also be factored in, as some participants may not have perceived dietary modification as a type of TCM.

We recommend that similar studies be conducted in different locations throughout the country, and they should preferably use probability sampling to improve on the aspect of generalizability. Future research may explore the association between TCM use and modes of delivery and engage in qualitative analyses to compare safe and unsafe TCM practices.

## Conclusions

Based on our findings, the prevalence of TCM use among postpartum women is high and varied, and it is strongly associated with ethnicity and household income. Most of the women use TCM to improve their general well-being and are influenced by their family beliefs. However, these findings should be interpreted with caution as they only represent the views of the population in the northern rural area of West Malaysia.

TCM has been practiced for a long time and it is here to stay as it has become more acceptable and mainstream. Therefore, it is time for medical doctors to familiarize themselves with these practices and be prepared to discuss TCM with patients.
